# Precarious employment and general, mental and physical health in Stockholm, Sweden: a cross-sectional study

**DOI:** 10.1177/1403494820956451

**Published:** 2020-09-16

**Authors:** Johanna Jonsson, Nuria Matilla-Santander, Bertina Kreshpaj, Gun Johansson, Katarina Kjellberg, Bo Burström, Per-Olof Östergren, Karin Nilsson, Susanne Strömdahl, Cecilia Orellana, Theo Bodin

**Affiliations:** 1 Unit of Occupational Medicine, Karolinska Institutet, Sweden; 2 Centre for Occupational and Environmental Medicine, Stockholm County Council, Sweden; 3 Centre for Epidemiology and Community Medicine, Stockholm County Council, Sweden; 4 Equity and Health Policy Research Group, Karolinska Institutet, Sweden; 5 Social Medicine and Global Health, Lund University, Sweden; 6 Global and Sexual Health, Karolinska Institutet, Sweden; 7 Section of Infectious Diseases, Uppsala University, Sweden

**Keywords:** Precarious employment, non-standard employment, occupational health, mental health, physical health

## Abstract

*Objectives:* To investigate the association between precarious employment and health in a sample of non-standard employees in Stockholm County, Sweden, by addressing three specific research questions: is the degree of precarious employment (low, moderate, high) associated with self-rated. . . (a) general health, (b) mental health, (c) musculoskeletal pain? *Methods:* Web-based respondent-driven sampling was used to recruit a sample of 415 employees in Stockholm, Sweden, during 2016–2017. Questionnaire data were collected on employment conditions (the Swedish version of the employment precariousness scale (EPRES-Se)), general health, mental health and musculoskeletal pain. EPRES-Se scores were categorised as low, moderate or high. Generalised linear models with Poisson distribution, log link functions and robust variances were applied for calculating crude and adjusted prevalence ratios (PR; aPR) with 95% confidence intervals (CIs) for all outcomes. *Results:* The prevalence ratios of poor self-rated general and mental health increased with increased degree of precariousness, as indicated by estimates of moderate precarious employment (a_2_PR_Moderate_ 1.44 (CI 0.98–2.11); a_2_PR_Moderate_ 1.13 (CI 0.82–1.62)), and high precarious employment (a_2_PR_High_ 1.78 (CI 1.21–2.62); a_2_PR_High_ 1.69 (CI 1.25–2.28)), albeit only significantly so for high precarious employment. ***Conclusions:* This is the first study in Sweden reporting on the association between precarious employment, as measured with a multidimensional scale, and multiple health outcomes. The results add to the evidence of an association between precarious employment and self-rated poor general and mental health. Larger, representative studies with longitudinal designs using the EPRES-Se are called for in order to strengthen these results and the already existing evidence of the harm of precarious employment.**

## Introduction

Precarious employment (PE) is a term attempting to encompass a range of attributes associated with (poor) employment quality. There is not yet consensus on a definition of PE that transcends sociopolitical and historical contexts [1]. However, PE is often characterised by temporariness in employment, income insufficiency, and a lack of labour/collective rights and social security [2]. As such, PE does not refer to the type of employment per se, such as temporary employment, but unidimensional measures of PE are nevertheless widely applied in research due to e.g. the lack of detailed labour market statistics on crucial PE features [3]. To our knowledge there is no reliable estimate of the extent of PE in the Swedish labour market. In the Swedish workforce approximately 35–39% are estimated to have an atypical employment (characterised by temporary employment, employment in a temp agency, self-employment, lack of collective bargaining agreement coverage, multiple jobs or work in the informal sector) [4], and 15–17% are estimated to be in temporary employment [5]. Together with Finland, Sweden has the highest prevalence of temporary employment in northern Europe [6]. Reports also show that the longer-term temporary positions in Sweden have been replaced with a larger proportion of on-demand employments and day labourers, making temporary employment even more precarious [4, 5].

### PE and health

PE is increasingly recognised as a social determinant of poor health, affecting both individuals as well as families and societies [7]. Epidemiological research has mainly made use of unidimensional operationalisations of PE, but several efforts have been directed towards using multidimensional operationalisations such as composite or summed scales [8–11], multivariable models including several PE indicators [12] and typological approaches [13–15].

A review assessing the health effects of insecure and precarious employment in different welfare regimes found mixed evidence for associations between PE (in most cases measured by employment type) and poor health. However, those in PE in Scandinavian welfare states presented better health outcomes in general, as compared with other welfare states [16]. A recent review and meta-analysis of longitudinal studies on mental health outcomes of PE also found inconsistent evidence for associations between temporary employment and poor health, while there were significant effects for multidimensional measures. All of the studies applying multidimensional measures of PE were from Scandinavian countries [17]. Cross-sectional studies assessing PE with the employment precariousness scale (EPRES) have reported associations between high employment precariousness and poor mental health [18–20]. Furthermore, a multivariable approach to PE has shown associations between low earnings, substantial unpaid overtime and benefit inadequacy and poor general and/or functional health outcomes, adjusting for all other factors. No significant associations between non-standard employment and either of the outcomes were found [12]. Finally, typological approaches to PE report PE types to be associated with mental distress/poor mental health [13], poor general health [14] and physical complaints, as well as protective effects for sick leave [15]. The association between PE and poor health has been suggested to operate through several pathways [12]. The first pathway being the experiences of PE; for example, feelings of unfairness or powerlessness, uncertainty about future employment, working times and income, etc. The second pathway is suggested to operate through social and material deprivation and the third by exposure to poor physical and psychosocial working conditions. The discussion around potential pathways, however, implies causation between PE and health. Here the risk of reverse causation must be considered. One example in which this relationship was established while accounting for reverse causation is a Swedish longitudinal study by Canivet et al. [21], supporting the hypothesis that PE influences poor health.

Overall, previous studies point towards an association between PE and poor health, transcending a range of multidimensional measures of PE as well as a range of outcomes. However, studies using comparable operationalisations of PE and studies investigating physical health outcomes such as musculoskeletal pain (MSP), are currently lacking. As such, the public health relevance of this study includes an expansion of the literature on the association between PE and self-rated general health, mental health and MSP in the Swedish context by assessing PE with the Swedish version of the employment precariousness scale (EPRES-Se) [9], facilitating future studies aimed at improving employment conditions and ultimately the health of workers in precarious conditions.

### Aim

The overall aim of this study was to investigate the association between PE and health in a sample of non-standard employees in Stockholm County, Sweden. More specifically, this study addressed the following three research questions: is the degree of PE (low, moderate, high) associated with self-rated. . .

(a) general health?
(b) mental health?
(c) MSP?

## Method

### Study design

This study has a cross-sectional design, using survey-based data on PE and self-reported health outcomes, in a sample of employees in non-standard employment arrangements. The current study was conducted within the project Precarious Employment in Stockholm (PREMIS), aimed at studying health outcomes of PE.

### Setting and participants

Data collection took place between November 2016 and May 2017. Inclusion criteria for the study were: working but not on a full-time permanent contract, living and/or working in Stockholm County, being 18–65 years of age and having a Swedish personal identification number. Exclusion criteria were: having permanent full-time employment, being voluntarily self-employed or voluntarily part-time employed, being a student or a pensioner. At the end of the data collection, 483 individuals were included in the sample. Out of these, 68 participants were excluded due to not matching the study criteria; that is, employment or county (*n*=6), re-using a personal number (*n*=8), giving an incorrect personal number (*n*=17), being under age (*n*=1) or due to suspected cheating (i.e. repeated participation) (*n*=36), yielding a final sample of 415 participants.

Participants were recruited using web-based respondent-driven sampling (WebRDS), which is a peer-to-peer recruitment strategy employed in hard-to-reach populations lacking a sampling frame. In respondent-driven sampling (RDS) methodology, the sample is weighted based on self-reported network size (degree) in order to compensate for oversampling individuals with large social networks; that is, overrepresentation of individuals likely to have similar characteristics [22]. Recruitment and collection of survey data was conducted with WebRDS software developed for the purpose. Further details on the recruitment can be found in a previous publication [23].

### Data sources

The survey (hereafter: PREMIS survey) included items on employment conditions (assessed with the EPRES-Se), work environment, health outcomes, current life situation and background. The survey could be completed in Swedish or English. The full survey, EPRES-Se, and further details on the data collection can be found in previous publications [9, 23]. Register data for the years 2016 and 2017 were obtained from the Register of the Total Population (RTB) and the Longitudinal Integration Database for Health Insurance and Labor Market Studies (LISA). Both registers are held by Statistics Sweden and contain individual level data on sociodemographic characteristics, such as age, sex, education, country of birth, occupation, etc. Data collected with the PREMIS survey were linked to LISA and RTB by Statistics Sweden by means of the personal identification number unique to every person registered in Sweden.

### Variables

#### Exposure variables

The degree of PE was assessed with the EPRES-Se, which consists of six dimensions and 23 items: ‘temporariness’ (contract duration and tenure; two items), ‘wages’ (low or insufficient and possible economic deprivation; three items), ‘disempowerment’ (level of negotiation of employment conditions; two items), ‘vulnerability’ (defenselessness to authoritarian treatment; five items), ‘rights’ (right to workplace rights and social security benefits; five items) and ‘exercise rights’ (powerlessness to exercise workplace rights; six items) [9]. Items are initially scored on a three- or five-point scale and thereafter converted to a 0–4 scale. Each dimension is averaged and thereafter a global average is calculated. Global scores theoretically range between 0 and 4, where 0 and 4 represent the lowest and highest scores of PE, respectively. For the purpose of obtaining the degree of PE in this study, EPRES-Se scores were divided into tertiles: low (0.9–1.66), moderate (1.67–2.12) and high (2.13–3.07).

#### Outcome variables

Self-reported outcomes on: (a) general health, (b) mental health [24], and (c) MSP were retrieved from the PREMIS survey. An overview of these can be found in Table I.

**Table I.** Overview of health outcomes and their operationalisation.

**General health**	*Question*: ‘How would you describe your health in general? Is it. . .’ ‘Very good’, ‘Good’, ‘Fair’, ‘Poor’, or ‘Very poor’ *Operationalisation*: Response options ‘Fair’, ‘Poor’ and ‘Very poor’ were categorised as less than good (i.e. poor) general health
**Mental health**	*Question battery*: The general health questionnaire with 12 items (GHQ-12) Scored with the GHQ-method (0–0–1–1) in which the two first response options receive 0, and the two final response options receive 1 *Operationalisation*: The total score theoretically ranges between 0 and 12, in which the cut-off for poor mental wellbeing (i.e. poor mental health) was three or more points
**MSP**	*Question*: ‘During the past 3 months after work have you had pain in. . . (a) upper back or neck? (b) lower back? (c) shoulders or arms? (d) wrists or hands? (e) hips, legs, knees or feet?’ and response options ‘Every day’, ‘A couple of days per week’, ‘One day per week’, ‘A couple of days per month’ or ‘Not at all/rarely in the last three months’ *Operationalisation*: Response options ‘Every day’, ‘A couple of days per week’ and ‘One day per week’ on a minimum of one location was used as an indicator of pain

MSP: musculoskeletal pain.

The operationalisation of the outcomes was made in accordance with previous literature with slight adaptations. In earlier dichotomous categorisations of general health, the response-scale option ‘fair’ has sometimes been left out, such as in reports from the Swedish Public Health Survey [25]. ‘Fair’ is more commonly grouped as poor health when the response options are ranked from ‘excellent’ to ‘poor’ (including ‘very good’, ‘good’, ‘fair’) [8, 13]. Due to limited power, however, leaving the option out was not an alternative. The cut-offs for mental health and MSP, respectively, were used in accordance with the Swedish Public Health Survey [25] and the Swedish Work Environment Survey [26]. In the latter, however, MSP was separated by location.

#### Covariates

Data on sex (male; female), age (continuous and categorised as 18–24, 25–29, 30–35 and 36–62 years), unemployment during the past three years (yes; no) and occupational social class (manual occupation; non-manual occupation) were collected from the PREMIS survey. Occupational social class was created by categorising self-reported current occupation into three-digit level SSYK 2012 codes (Swedish abbreviation for Swedish Standard Classification of Occupations for 2012, which is a modification of ISCO-08), thereafter grouping these on a one-digit level ranging from 1 to 9, and finally dividing them into manual and non-manual occupations (level 5–9 and 1–4, respectively [27]). From LISA, data were collected on highest completed education (categorised as high school; higher education ≤2 years; or higher education ≥3 years) and family composition (married/cohabiting with or without children living at home; single with children living at home; single without children). From RTB, data were collected on country of birth (Sweden; outside of Sweden). Data on education and family composition were matched on participation year in the PREMIS survey. The sufficient adjustment variables, as described above, for estimating the total effect of PE on the health outcomes were identified through the construction of a directed acyclic graph (DAG) for each of health outcome using ‘DAGitty’ [28], see Supplemental Figures 1–3 in the Supplemental material.

### Statistical methods

Frequencies with 95% confidence intervals (CIs) were calculated for all covariates according to the degree of precariousness. The prevalence (with CIs) of each health outcome was calculated according to the degree of precariousness and presented in a bar graph. Generalised linear models with Poisson distribution, log link functions and robust variances were applied for calculating the crude and adjusted prevalence ratios (PR; aPR) with 95% CIs for all outcomes [29]. Models were constructed by including confounders in two steps: first by including sex, age (continuous), education and occupational social class, and secondly adding country of birth, previous unemployment and family composition. A low degree of PE was used as reference in all models.

In addition to the main analyses, a number of sensitivity analyses were performed. First, as MSP differed from general health and mental health in the DAGs in the sense that it was not necessary to adjust for previous unemployment, the association between PE and MSP was adjusted for unemployment in the main analysis, while a sensitivity analysis was conducted without previous unemployment. Secondly, in order to accommodate the potential limitation that might arise from the poor psychometric properties of the temporariness dimension in EPRES-Se [9] crude and adjusted models were conducted without this dimension. Finally, frequencies as well as crude and adjusted models were weighted in accordance with RDS methodology. Weighted results are presented in the Supplemental material. RDS-II weights were calculated in RDS-Analyst 0.42 for Windows (Los Angeles, CA, USA). Unweighted analyses were conducted using statistical analysis software (SAS) and weighted analyses were conducted using STATA 14.0.

### Ethics statement

The study was approved by the regional ethics board of Stockholm (2016/1291-31/5). All participants gave written informed consent before participating in the study, by clicking ‘yes’ to the question ‘I understand the information given above and want to participate’ after reading the study information. Collected data were stored on password encrypted servers and personal identification numbers were replaced by serial numbers. The key for the latter is held by Statistics Sweden and was inaccessible to the researchers.

## Results

**Figure 1.**
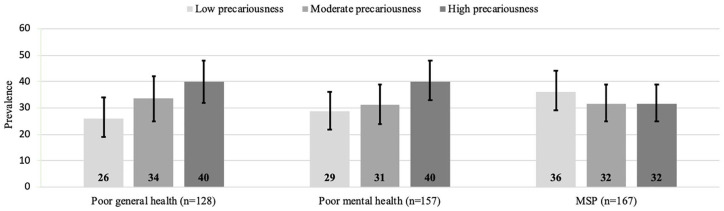
Prevalence (95% CIs) of health outcomes stratified by degree of precariousness.

Table II shows the sociodemographic characteristics of the sample, overall and stratified by the degree of precariousness. The participants in the group with a high degree of precariousness, as compared to the group with a low degree of precariousness, were to a larger extent men (51.8% vs. 42.4%), younger than 25 years (43.9% vs. 13.6%), had more often only attained a high school degree as highest education (49.6% vs. 34.6%), had more often experience of previous unemployment (57.5% vs. 37.1%) and were more often currently working in manual occupations (66.4% vs. 49.6%). In addition, a larger proportion of those with a high degree of precariousness were born outside Sweden (29.0%), compared to those with a low degree of PE (13.7%). Most of these frequencies increased with increased precariousness in a gradient fashion. Both poor self-rated general health and mental health increased in prevalence with increased precariousness, with prevalences of 40%, respectively, for participants in the highest degree of precariousness. The reverse could be seen for MSP, in which the prevalence was the largest, with 36% in the group with low precariousness. See Figure 1.

**Table II.** Sociodemographic characteristics of the sample, overall and stratified by degree of precariousness (*n*=401).

	Low precariousness			Moderate precariousness			High precariousness			Total	
	*n*	%	95% CI	*n*	%	95% CI	*n*	%	95% CI	*n*	%
**Sex**											
Men	56	42.4	34.0–50.9	56	43.1	34.6–51.6	72	51.8	43.5–60.1	184	45.9
Women	76	57.6	49.1–66.0	74	56.9	48.4–65.4	67	48.2	39.9–56.5	217	54.1
Total	132	100		130	100		139	100		401	
**Age**											
18–24	18	13.6	7.8–19.5	40	30.8	22.8–38.7	61	43.9	35.6–52.1	119	29.7
25–29	67	50.8	42.2–59.3	54	41.5	33.1–50.0	55	39.6	31.4–47.7	176	43.9
30–35	25	18.9	12.3–25.6	28	21.5	14.5–28.6	8	5.8	1.9–9.6	61	15.2
36–62	22	16.7	10.3–23.0	8	6.2	2.0–10.3	15	10.8	5.6–15.9	45	11.2
Total	132	100		130	100		139	100		401	
**Education**											
High school	45	34.6	26.4–42.8	47	36.4	28.1–44.7	64	49.6	41.0–58.2	156	40.2
Higher education, ≤2 years	30	23.1	15.8–30.3	30	23.3	15.9–30.6	35	27.1	19.5–34.8	95	24.5
Higher education, ≥3 years	55	42.3	33.8–50.8	52	40.3	31.9–48.8	30	23.3	15.9–30.6	137	35.3
Total	130	100		129	100		129	100		388	
**Country of birth**											
Sweden	113	86.3	80.4–92.0	111	85.4	79.3–91.5	98	71.5	63.9–79.1	322	80.9
Outside Sweden	18	13.7	7.9–19.6	19	14.6	8.5–20.1	39	28.5	20.9–36.0	76	19.1
Total	131	100		130	100		137	100		398	
**Previous unemployment**											
Yes	49	37.1	29.0–45.4	63	48.8	40.2–57.5	80	57.5	49.3–65.8	192	48.0
No	83	62.9	54.6–71.1	66	51.2	42.5–59.8	59	42.5	34.2–50.7	208	52.0
Total	132	100		129	100		139	100		400	
**Family composition**											
Single	75	57.7	49.2–66.2	79	60.8	52.4–69.2	78	57.8	49.5–66.1	232	58.7
Single with children	20	15.4	9.2–21.6	21	16.2	9.8–22.5	24	17.8	11.3–24.2	65	16.5
Married/cohabiting w/wo children	35	26.9	19.3–34.6	30	23.1	15.8–30.3	33	24.4	17.2–31.7	98	24.8
	130	100		130	100		135	100		395	
**Occupation**											
Manual	61	49.6	40.8–58.4	58	45.7	37.0–54.3	91	66.4	58.5–74.3	210	54.3
Non-manual	62	50.4	41.6–59.2	69	54.3	45.7–63.0	46	33.6	25.7–41.5	177	45.7
Total	123	100		127	100		137	100		387	

CI: confidence interval.

In Table III the crude and adjusted PRs with 95% CIs are presented for all outcomes. The fully adjusted PR of poor self-rated general health increased with increased precariousness in a gradient manner (a_2_PR_Moderate_ 1.44 (CI 0.98–2.11); a_2_PR_High_ 1.78 (CI 1.21–2.62)), although the estimate for moderate precariousness was not significant. A similar pattern was seen for poor self-rated mental health (a_2_PR_Moderate_ 1.13 (CI 0.82–1.62); a_2_PR_High_ 1.69 (CI 1.25–2.28)). There were no observed associations between PE and MSP. These results were also observed in the partially adjusted models, with the exception of poor general health in which also moderate PE showed significant estimates (a_1_PR_Moderate_ 1.49 (CI 1.02–2.18)).

**Table III.** Prevalence ratios with 95% CIs for general health, mental health and MSP.

	Poor general health		Poor mental health		MSP	
**Crude model**	PR	95% CI	PR	95% CI	PR	95% CI
Low precariousness	1		1		1	
Moderate precariousness	1.28	0.88–1.88	1.11	0.80–1.53	0.90	0.68–1.18
High precariousness	1.43	0.99–2.05	1.33	0.99–1.79	0.83	0.63–1.10
**Adjusted model 1** ^a^						
Low precariousness	1		1		1	
Moderate precariousness	1.49	1.02–2.18	1.11	0.81–1.52	1.00	0.78–1.28
High precariousness	1.78	1.22–2.59	1.62	1.21–2.17	1.09	0.82–1.44
**Adjusted model 2** ^b^						
Low precariousness	1		1		1	
Moderate precariousness	1.44	0.99–2.11	1.13	0.82–1.55	0.98	0.77–1.24
High precariousness	1.78	1.20–2.62	1.69	1.25–2.28	1.06	0.78–1.41

CI: confidence interval.

a Adjusted for sex, age (continuous), education and occupational social class.

b Adjusted for sex, age (continuous), education, occupational social class, previous unemployment, birth country and family composition.

Results from the sensitivity analysis using weights showed small differences in weighted frequencies of sociodemographic characteristics, as compared to the unweighted frequencies (5–13 percentage points for a few variables), and did not change the overall interpretation (see Supplemental Table I). The significant estimates for poor self-rated general health and mental health remained when weighted, although with larger estimates (see Supplemental Table II). Excluding the temporariness dimension generally confirmed the results stemming from the full EPRES-Se. However, the magnitude of some of the estimates changed: the PR for poor self-rated general health increased and turned significant for moderate PE (a_2_PR_Moderate_ 1.51 (CI 1.02–2.21)), as well as increased among high PE (a_2_PR_High_ 1.93 (CI 1.29–2.88)); while the PR was slightly reduced for poor mental health in the high PE group (a_2_PR_High_ 1.57 (CI 1.77–2.11)) (data not shown). Removing previous unemployment from the fully adjusted model of PE and MSP had minor influence on the estimates (a_2_PR_Moderate_ 0.99 (CI 0.78–1.27); a_2_PR_High_ 1.12 (CI 0.84–1.49)) and did not change the interpretation of the results.

## Discussion

This is the first study in Sweden reporting on the association between PE, as measured with a multidimensional scale, and multiple health outcomes. It is also one of few studies assessing MSP as an outcome. The results showed positive associations between PE and both poor self-rated general health and mental health.

### General health and mental health

The association between PE and poor self-rated general health found in this study is in line with previous studies. One similar example is a Spanish study on Catalonian workers using the EPRES. This study, however, found a clearer tendency for a gradient association between EPRES quartiles and self-rated general health, as well as larger estimates and particularly so among men (4th quartile PR of 2.69 (CI 1.62–4.49) and 2.14 (CI 1.34–3.43) among women, as compared with a 3rd tertile PR of 1.78 (CI 1.20–2.62) in the current study) [19]. The finding of an association between PE and poor self-rated mental health has also been supported by previous studies from Spain using the EPRES (including the study on Catalonian workers) [18, 19]. Both of these studies also found clearer gradient patterns of poor mental health as well as larger estimates (particularly among women), as compared with the current study (the study on Catalonian workers found 4th quartile PRs of 3.45 (CI 2.11–5.65) for women and 3.21 (CI 2.08–4.95) for men, the Spanish study reported 5th quintile prevalence proportion ratios (PPRs) of 2.54 (CI 1.95–3.31) for women and 2.23 (CI 1.86–2.68) for men, while the current study found a 3rd tertile PR of 1.69 (CI 1.25–2.28)). There are several reasons why the results could differ. For one, Sweden and Spain are different in terms of welfare state regimes. It has previously been reported that PE in southern welfare states is associated with a higher risk of health complaints and mental illness, as compared with permanent employment; while precarious employees in Scandinavian welfare states present with better health outcomes. This moderating effect might be due to the more comprehensive employment policies of Scandinavian welfare states [16]. Spain, compared to Sweden, also suffered higher unemployment rates both before and after the economic crisis of 2008, contexts in which these studies were conducted [10, 18, 30]. This bears importance as high unemployment rates are inherently linked with PE conditions [10]. Furthermore, the samples of the Spanish studies were representative of the population (including both standard and non-standard employees) and larger in size, which allow for more precise estimates in addition to finer categorisation of PE. These factors are all likely to have contributed to the associations being stronger in the Spanish data, in comparison with the Swedish data.

Setting the studies using the EPRES aside, the results of this study are also supported by other studies. For instance, studies operationalising PE with a summative score report a gradual increase in the prevalence of poor self-reported health with increasing precariousness [11], and higher odds of poor general health if precarious [8]. Further examples are taken from the typological approaches; that is, studies grouping employment characteristics with the use of latent class analysis and identifying typical PE types. Using US data, the associations of PE types with poor self-rated general health were significant in crude models [13], while the associations were significant for both crude and adjusted models in European data [14, 15]. In terms of mental health, both typological and summative score approaches confirm the association between PE and poor self-rated mental health [8, 14, 15].

### Musculoskeletal pain

The results of this study provide no evidence for an association between PE and MSP. Although there is a scarcity of studies on this association, one study has assessed this outcome in 35 European countries [11] and one among bus drivers and conductors in Brazil [31]. Both of these studies report a higher prevalence of MSP among precarious employees in the 3rd and 4th quartiles (in the Brazilian study, the 4th quartile PR was 1.24 (1.04–1.46), while the 3rd tertile PR was 1.06 (CI 0.78–1.41) in the current study. The European study only presented estimates in figures). Despite the lower of the 3rd and 4th quartiles being non-significant, the PRs followed a gradient pattern [11, 31]. One explanation for the results found in the current study, could be that physically strenuous work is more common among both young and manual workers, while pain conditions, however, are more common among older age groups in the Swedish workforce [26]. In this study, the high precariousness group was characterised by a large proportion of young and manual workers, who might not yet have developed severe pain conditions; while the low PE group was characterised by larger proportions of older workers. Furthermore, if the work involves heavy physical work, constrained work postures and/or repetitive work, there is also a possibility that PE may protect against MSP. If the precarious conditions imply that the workers often are changing employment and work tasks, this may entail more variation in physical exposures, in comparison with workers in permanent employment exposed to similar physical exposures all the time. Variation in biomechanical exposure has been proposed as a preventive factor for musculoskeletal disorders [32, 33]. Finally, it is also possible that a healthy worker effect is in play, where physically healthy workers to a greater extent remain in precarious conditions. All of these aspects could potentially lead to a dilution of effects and hence a lack of observable patterns. Due to the cross-sectional nature of this study, however, such conclusions cannot be drawn with certainty.

### Strengths and limitations

This is one of few studies exploring the potential gradient association between PE and health outcomes, and in particular with MSP, as well as the first study assessing these health outcomes with the EPRES-Se. There are, however, some limitations to this study. First, the sample size is relatively small and did not allow for stratification by hypothesised effect modifiers, such as sex and age, nor did it allow for finer categorisation than tertiles, as compared with previous studies. Secondly, this being a cross-sectional study gives rise to the potential of reverse causation; that is, that the experienced poor health conditions (i.e. outcomes) influences the degree of precariousness (i.e. exposure). This is in line with a potential ‘healthy hire effect’ and ‘healthy worker survivor effect’ in which healthy individuals are hypothesised more often to be selected in to (permanent) employment and to remain in employment, as compared with unhealthy individuals (who thus suffer greater risks of ending up in PE or unemployment) [34]. These hypotheses were partly supported in a study on employment contract trajectories from The Netherlands [34]. However, in a PE research context, single measure indicators of PE, such as employment contract, have been argued against while multidimensional constructs repeatedly have been encouraged in order to capture the full extent of PE (e.g. Bodin et al. [1] and Benach et al. [7]). Furthermore, contract type has inconsistently been associated with poor health in previous research. For instance, a meta-analysis of longitudinal studies of PE and mental health found strong effects in terms of multidimensional operationalisations but inconsistent effects for temporary employment [17]. This supports the hypothesis that (multidimensional) PE indeed can influence poor health, although more rigorous longitudinal evidence is needed in the respect of reverse causality.

Furthermore, the sample being a convenience sample limits the generalisability of the results. In addition, the sample only included non-standard employees, while the EPRES was developed to assess permanent and temporary employees alike. This could have contributed to a dilution of the estimated effects, as permanent (‘standard’) employees are more likely to have a lower degree of precariousness [20]. In addition, the survey could only be completed in Swedish or English, which could have excluded parts of the foreign-born precarious population with insufficient language skills. Hence, we might expect stronger associations with a larger, representative sample. The fact that this study in essence confirmed results from previous studies using EPRES on the general working population, both in the unweighted and weighted samples, indicates that the sample could be used to support previous literature in terms of general and mental health outcomes.

## Conclusions

This study adds to the evidence of an association between PE and poor self-rated general and mental health. Future studies are warranted in Sweden to apply the EPRES-Se in relation to these (and other) health outcomes in larger and representative samples of the general population, especially in terms of MSP, in order to clarify whether an association exists. As sex and age could be important effect modifiers in these associations, the continued use of stratified analyses is called for. Finally, longitudinal studies are necessary in order to exclude the risk of reverse causation and strengthen the already existing evidence of the harm of PE.

## Supplemental Material

SJP956451_Supplemental_material – Supplemental material for Precarious employment and general, mental and physical health in Stockholm, Sweden: a cross-sectional study Click here for additional data file. Supplemental material, SJP956451_Supplemental_material for Precarious employment and general, mental and physical health in Stockholm, Sweden: a cross-sectional study by Johanna Jonsson, Nuria Matilla-Santander, Bertina Kreshpaj, Gun Johansson, Katarina Kjellberg, Bo Burström, Per-Olof Östergren, Karin Nilsson, Susanne Strömdahl, Cecilia Orellana and Theo Bodin in Scandinavian Journal of Public Health
