# Supplementary material for: Precarious employment and general, mental and physical health in Stockholm, Sweden: a cross-sectional study
Source: Scand J Public Health. 2020 Sep 16;49(2):228–36. doi: 10.1177/1403494820956451 (PMC7917568; doi:10.1177/1403494820956451)

Table S1. Weighted sociodemographic characteristics of the sample, overall and stratified by degree of precariousness							
	**Low precariousness**		**Moderate precariousness**		**High precariousness**		**Total**
	%	95 % CI	%	95 % CI	%	95 % CI	%
**Sex**							
Men	48.9	38.5-59.3	47.6	36.9-58.6	55.2	44.9-64.9	50.7
Women	51.1	40.6-61.5	52.4	41.4-63.1	44.8	35.1-55.1	49.3
**Age**							
18-24	16.1	9.5-25.9	35.2	25.5-46.4	48.1	38.1-58.2	33.7
25-29	48.4	38.1-58.9	40.6	30.6-51.6	38.9	29.8-49.1	42.5
30-35	16.6	9.8-26.5	20.8	12.9-31.7	2.6	1.19-5.6	13
36-62	18.9	11.6-29.3	3.3	0.96-11.1	10.4	5.3-19.3	10.8
**Education**							
High school	38.9	29.2-49.6	42.3	31.8-53.5	57.1	46.6-67	46.2
Higher education ≤2 y	27.4	18.7-38.1	21.4	13.7-31.7	19.5	13.3-27.6	22.7
Higher education ≥3 y	33.7	24.3-44.6	36.3	26.5-47.5	23.5	15.3-34.3	31.1
**Country of birth**							
Swedish	82.6	72.4-89.5	84.4	75.1-90.7	72.5	62.6-80.5	79.6
Non-Swedish	17.4	10.5-27.6	15.5	9.3-24.9	27.5	19.4-37.4	20.4
**Previous unemployment**							
Yes	23.8	16.3-33.2	39.4	29.5-50.4	53.5	43.2-63.4	39.4
No	76.2	66.7-83.7	60.5	49.6-70.5	46.5	36.6-56.8	60.5
**Family composition**							
Single	52.1	41.5-62.6	57.8	47.5-67.6	57.8	47.5-67.6	55.9
Single w. children	13.9	8.4-22.1	17.6	11.1-26.7	17.6	11.1-26.7	17.1
Married/cohabiting w/wo children	33.9	24.4-45.1	24.6	16.9-34.3	24.6	16.9-34.3	27.1
**Occupational social class**							
Manual	55.6	44.5-66.2	52.3	41.2-63.2	74.2	64.9-81.7	61.3
Non-manual	44.4	33.8-55.5	47.7	36.8-58.8	25.8	18.3-35.1	38.7

Table S2. Weighted prevalence ratios with 95% CI's for general health, mental health and pain

	**General health**		**Mental health**		**MSP**	
**Crude model**	*PR*	95 % *CI*	*PR*	95 % *CI*	*PR*	95 % *CI*
Low precariousness	1		1		1	
Moderate precariousness	1.27	0.72-2.24	1.10	0.68-1.77	0.71	0.45-1.11
High precariousness	1.63	0.98-2.72	1.50	0.99-2.27	0.89	0.60-1.31
**Adjusted model 1[a]**						
Low precariousness	1		1		1	
Moderate precariousness	1.81	1.03-3.2	1.13	0.71-1.79	0.85	0.55-1.31
High precariousness	2.49	1.47-4.22	1.87	1.27.2.77	1.28	0.88-1.86
**Adjusted model 2[b]**						
Low precariousness	1		1		1	
Moderate precariousness	1.69	0.95-3.01	1.20	0.75-1.94	0.80	0.54-1.18
High precariousness	2.38	1.35-4.18	2.03	1.34-3.09	1.15	0.75-1.76

[a] Adjusted for sex, age (continuous), education and occupational social class

[b] Adjusted for sex, age (continuous), education, occupational social class, previous unemployment, birth country and family composition

Figure S1. DAG of precarious employment and general health

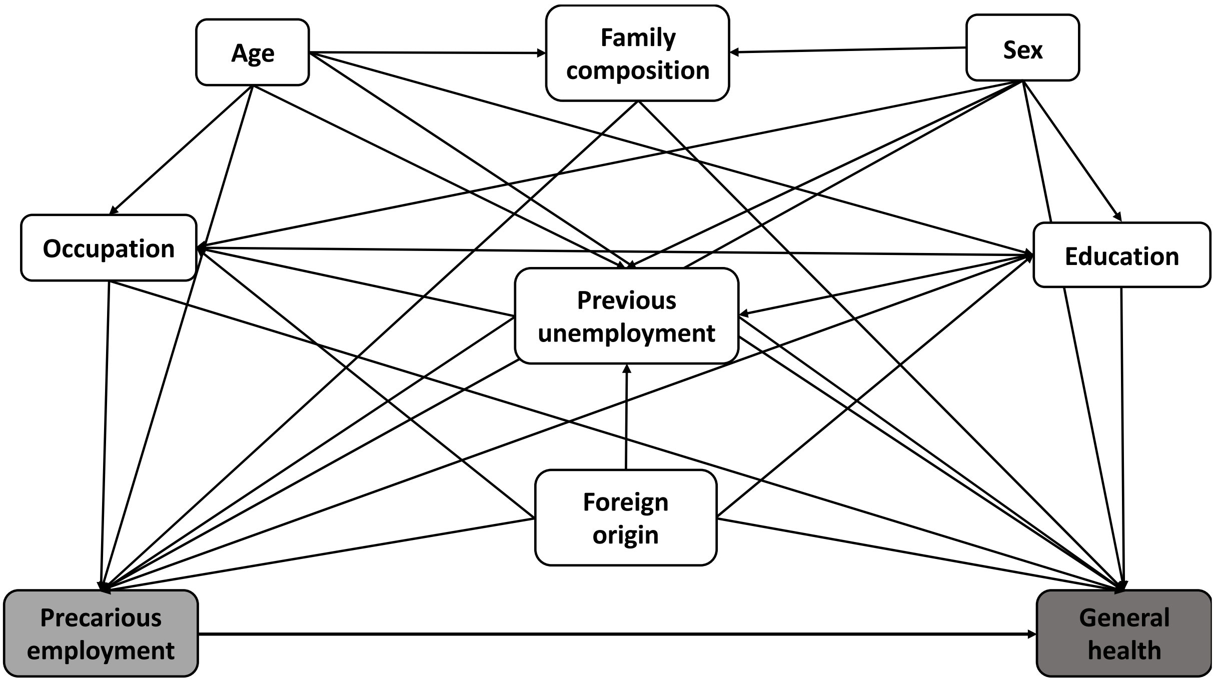

Figure S2. DAG of precarious employment and mental health

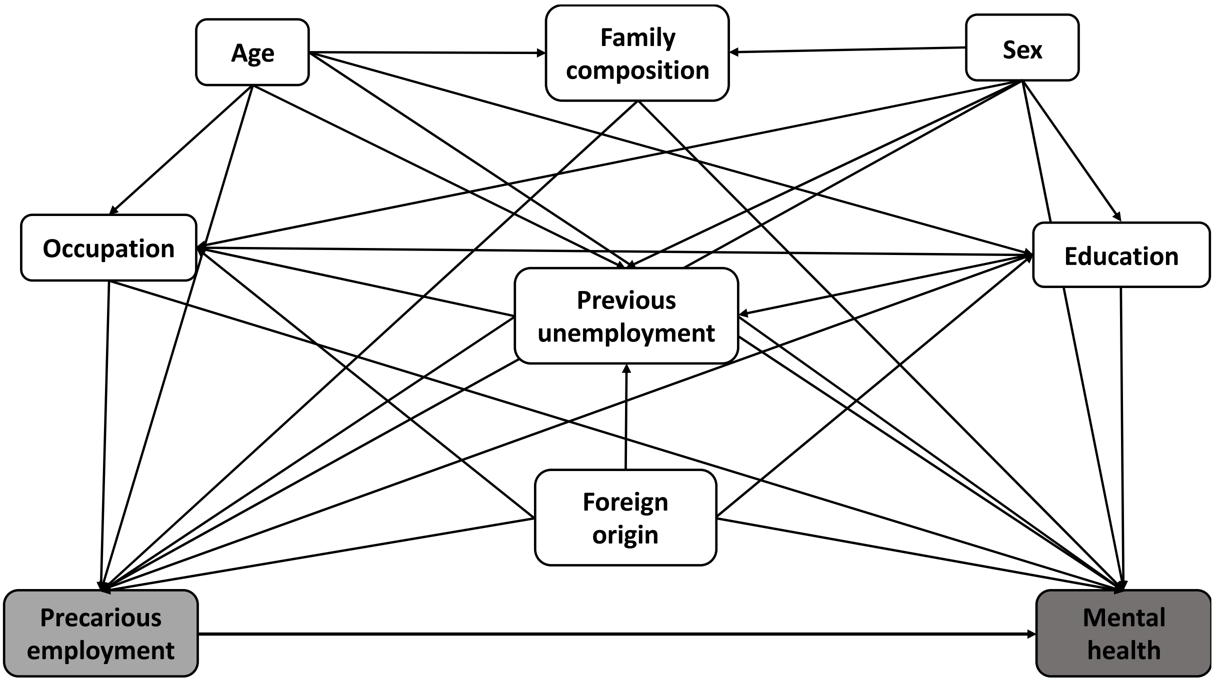

Figure S3. DAG of precarious employment and MSP

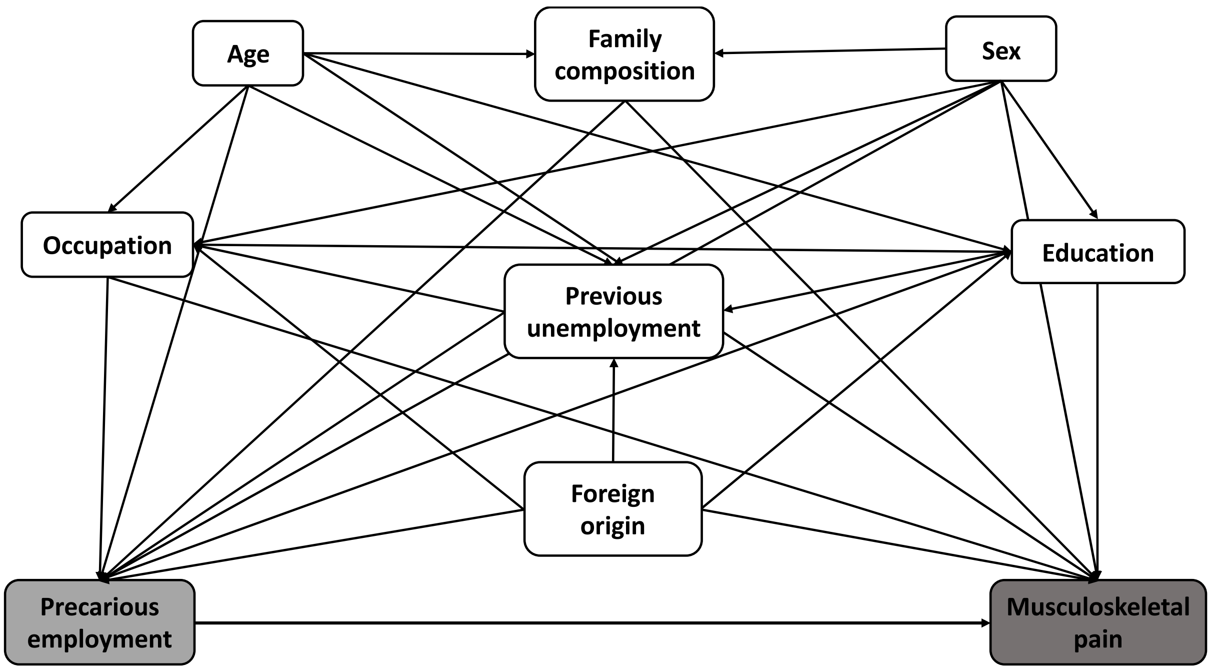

Supplement: SJP956451_Supplemental_material – Supplemental material for Precarious employment and general, mental and physical health in Stockholm, Sweden: a cross-sectional study [file SJP956451_Supplemental_material.pdf]
